# The effect of different sources of mesenchymal stem cells on microglia states

**DOI:** 10.3389/fnagi.2023.1237532

**Published:** 2023-08-24

**Authors:** Qiang Xin, Wenhao Zhu, Chuan He, Tianyi Liu, Haifeng Wang

**Affiliations:** Department of Neurotrauma Surgery, The First Hospital of Jilin University, Changchun, China

**Keywords:** mesenchymal stem cells, microglia, states, different sources, effect

## Abstract

Microglial reaction plays a key role in the prognosis of traumatic CNS injuries (TBI and SCI). A growing number of studies have shown that mesenchymal stem cells (MSCs) play an important role in regulating microglial states. This review summarizes the effects and mechanisms of different sources of MSCs on microglial states in the last 5 years. In general, bone marrow-derived mesenchymal stem cells are the most accessible and widely used, and can produce immunosuppressive effects on a variety of brain injuries including TBI through tissue engineering *in situ* implantation; MSCs mainly regulate inflammatory pathways and promote the states of microglia in the anti-inflammatory direction, which also secrete certain cytokines or extracellular vesicles to affect apoptotic pathways, such as the extracellular vesicles miR-21-5p, acting as a neuronal protector.

## Introduction

Traumatic CNS injuries include traumatic brain injury (TBI), spinal cord injury (SCI), and so on. TBI, according to the Centers for Disease Control and Prevention, is a disruption of normal brain function caused by a blow, strike, bump, or penetrating injury to the head (Capizzi et al., 2020). Globally, approximately 70 million people suffer from TBI each year, resulting in significant physical and psychological harm and economic burden to patients (Wiles, 2022). Neurological impairment after brain injury includes cognitive impairment, coma, sensory and motor impairment, visual impairment, and language impairment (McKee and Lukens, 2016). SCI refers to structural or functional spinal cord damage that occurs following damage to the spinal cord from a variety of causes, resulting in permanent loss of movement and sensation below the plane of injury. TBI and SCI are divided into primary and secondary injuries, with transient mechanical external forces acting on the skull causing primary injury while secondary injury results in a series of pathophysiological responses of the body in response to primary injury (Xiong et al., 2013). In the absence of exogenous infection, primary injury is followed by a sterile neuroimmune and neuroinflammatory response (Corps et al., 2015). In contrast, secondary injuries are characterized by glial cell activation, leucocyte recruitment, and upregulation of inflammatory mediators (Xiong et al., 2018). In addition to traumatic CNS injuries, traumatic subarachnoid hemorrhage combined with cerebral contusion, occlusion of major cerebral blood vessels, and stroke can also cause severe microglial reactions. Traditional treatments for severe craniocerebral injuries include debridement decompression, prophylactic sub-hypothermia, hyperosmotic therapy, cerebrospinal fluid drainage, and ventilation therapy (Galgano et al., 2017). Traditional treatments for SCI include surgical decompression, therapeutic hypothermia, and drugs targeting inflammation or excitotoxicity (Ramer et al., 2014). Despite significant advances in these treatments for traumatic CNS injuries, mortality rates for patients following traumatic CNS injuries remain high and their neurologic damage is not effectively repaired. In contrast, tissue engineering approaches using MSCs seem to have broader prospects. The results from some preclinical studies have shown that transplantation of bone marrow-derived mesenchymal stem cells (BMSCs) reduces secondary neurodegeneration and neuroinflammation, promotes neural and vascular neogenesis, and improves functional prognosis in experimental animals (Li J. R. et al., 2021).

Mesenchymal stem cells (MSCs) are pluripotent stem cells that have all the common properties of stem cells, namely, self-renewal and multidirectional differentiation capabilities. Now found in almost all tissues of the human body, the pluripotency of MSCs allows them to differentiate into osteoblasts, chondrocytes, adipocytes, or other lineages (Wang et al., 2021). MSCs exhibit immune tolerance by influencing innate and adaptive immune responses. This is reflected in the ability of MSCs to suppress immune cell responses by secreting immunomodulatory factors as well as to promote an immunosuppressive environment (Fernandes et al., 2020). MSCs slow down inflammatory injury in the central nervous system by interacting with macrophages/microglia (Harrell et al., 2019). Microglia, as resident immune cells of the CNS, are involved in the secondary inflammatory response after TBI together with macrophages of peripheral origin (Woodcock and Morganti-Kossmann, 2013).

The two states of macrophages include classically activated M1 and alternatively activated M2 phenotypes (Yunna et al., 2020). Microglia have a similar state process during CNS injuries (Hu et al., 2012). The traditional classification of the states of microglia responding to inflammation is M1 and M2 (Table 1). M1 macrophages/microglia are mainly involved in proinflammatory responses, and the M2 phenotype are mainly involved in anti-inflammatory responses. M1 macrophages/microglia usually release proinflammatory factors, including TNF-α, CCL2, and IL-6, which induce neuroinflammatory and toxic effects; in contrast, M2 macrophages/microglia secrete anti-inflammatory factors, such as IL-4, and neurotrophic factors, such as insulin growth factor (IGF), to exert anti-inflammatory repair effects (Li M. et al., 2021). Another method of categorization is to classify M2-type microglia into M2a, M2b, and M2c. They think the normal wound repair process is the sequential activation of classically activated M1-type and alternatively, activated M2a, M2b, and M2c-type macrophages to achieve structural and functional recovery of the injured tissue. However, nerve repair after TBI is difficult not only because neurons are permanent cells, but the excessive inflammatory response present at the site of injury is also an important factor that hinders nerve repair. The process of wound repair consists of three phases: inflammation, proliferation, and remodeling. The pro-inflammatory markers IL-12, IL-1β, TNF-α, and IL-6 increased dramatically in the early post-TBI period, and microglia in this phase also expressed high levels of arginase (Arg) and Ym-1, suggesting that the inflammation phase consisted of microglia of M1 and M2a phenotypes. The M2 microglia in the proliferation phase have different phenotypic characteristics from the M2 microglia in the inflammation phase. The expression of IL-10 is a hallmark of the M2b phenotype, which is secreted by the M2b microglia to promote tissue remodeling and thus plays a crucial role in the proliferation phase. It has been shown that the M2b microglia phenotype is improperly activated after SCI. The remodeling phase of normal wound healing is mainly dominated by M2c macrophages, which exhibit high expression of TGF-β and CD206, along with a decrease in Arg-1 (Gensel and Zhang, 2015). MSCs achieve nerve repair by promoting sequential activation of microglia after TBI. Extracellular vesicles were extracted from the supernatant of BMSCs cell culture, and the TBI mouse model was induced after tail vein injection. It was demonstrated that BMSCs promoted the expression of Arg-1, IL-10, and TGF-β after TBI by secreting the extracellular vesicles miR-181b (Wen et al., 2022), suggesting that MSCs can promote the expression of all the subtypes of M2-type microglia (Li et al., 2017), and in particular, promote the effective states of M2b and M2c.3 d and 7 d after TBI, we measured the expression of TNFα, CD86, Ym-1, Arg-1, and CD206 mRNA expression, and the results showed that BMSCs implanted at the injury site were effective in promoting microglia states to M2a and M2c (Zanier et al., 2014). Subtype differentiation of MSCs was found *in vitro* under exposure to stimuli, however, the complex environment *in vivo* may not apply to these states of differentiation. Paolicelli et al. (2022) put forward describe microglia using as many layers of complexity as possible and do not use M1 versus M2 classification. The following description of MSCs utilizes recommendations made by Paolicelli et al. (2022).

**TABLE 1:** The markers, roles, and secreted cytokines of M1 and M2 microglia.

Cell type	Markers	Cytokines	Effect	References
M1	CD86, MHC-II, CD11b, CD11c, CD14, CD16, CD32, CD36, CD40, CD45, CD47, FcR	TNF-α, iNOS, IL-6, IL-12 IL-17, IL-18, IL-23, IL-1βIFN-γ, TNF-a, CCL5 CCL20, CXCL1, CXCL9 CXCL10, MMP-9, MMP-12	Secretion of a variety of pro-inflammatory factors involved in pathogen clearance and reduction of inflammatory damage in disease. Excessive activation exacerbates the inflammatory response in the central nervous system.	Cherry et al., 2014; Jurga et al., 2020; Ellwanger et al., 2021; Guo et al., 2022; Wang et al., 2023
M2	CD206, CD163	IL-4, IL-10, IL-13, IGF-1 TGF-β Arg-1, FGF, CSF-1 NGF, FGF, BDNF, GDNF CSF-1, NT, PGRN, FIZZ-1 Ym-1, CCL2, CCL17 CCL22, CCL24	Involved in the repair of the central nervous system after injury, with anti-inflammatory and immunosuppressive effects.	

Mesenchymal stem cells can effectively suppress microglial reaction after TBI and SCI by regulating CNS microglial states and have a wide range of applications. However, it is not clear how the implanted MSCs regulate microglial states, so further studies on the regulatory mechanisms of MSCs are demanded to provide new ideas for neural repair after traumatic CNS injuries.

## Mechanisms by which mesenchymal stem cells regulate microglial states

In the early stages after traumatic CNS injuries, microglia create a favorable microenvironment for tissue regeneration by removing tissue debris (Devanney et al., 2020; An et al., 2021). In contrast, few reparative microglia are induced after TBI (Kigerl et al., 2009). Therefore, using a tissue engineering approach after the beneficial effects of the microglia would facilitate neural repair in TBI.

## MSCs regulate microglial states through a paracrine mechanism of cytokines

Studies have shown that MSCs can promote recovery from neurologically related diseases by secreting a variety of soluble factors, including trophic factors and cytokines (Teixeira et al., 2017). It has been suggested that the prolonged presence of microglial reaction after TBI increases the chance of Alzheimer’s disease (Ramos-Cejudo et al., 2018). In studies of Alzheimer’s disease models, transplanted MSCs were found to promote the conversion of microglia from the CD86^+^ to the Arg-1^+^ state through paracrine effects (Campos et al., 2022; Zhang R. et al., 2022). MSCs regulate microglial states by secreting the paracrine factors Galectin-1 (Gal-1) (Seo et al., 2022), tumor necrosis factor-alpha-induced gene/protein 6 (TSG-6) (Liu et al., 2019; Nam et al., 2020), and IL-13 (Taj et al., 2018), etc. Promoting microglial states toward the anti-inflammatory phenotype facilitates the relief of deafferentation pain. MSCs may regulate the functional state of microglia by activating the STAT6 signaling pathway, decreasing the expression of proinflammatory cytokine and promoting the expression of anti-inflammatory cytokine (Liu et al., 2022). MSCs, stimulated with TNF-α, regulate microglial activity by secreting TNF-α stimulated gene/protein 6 (TSG-6), which may interact with CD44 on the surface of microglia and inhibit LPS-induced activation of the TLR4 signaling pathway in microglial cells. Inhibition of the TLR4 signaling pathway impeded activation of the nuclear factor (NF)-κB and mitogen-activated protein kinase (MAPK) signaling pathways, thereby reducing the expression level of neuroinflammatory cytokines in BV2 microglia (Liu et al., 2014).

Homeostatic-state microglia do not express iNOS and Arg-1, but the stimulation of LPS upregulates iNOS expression and decreases Arg-1 expression. In a preclinical study, BMSCs were found to affect the miR-30a-3p/XBP1/MANF pathway in microglia by secreting platelet-derived growth factor-AA (PDGF-AA). Specifically, the PDGF-AA, secreted by BMSCs, downregulates miR-30a-3p in microglia. The decline of miR-30a-3p can enhance X-box binding protein (XBP) 1 expression, leading to increased expression of mesencephalic astrocyte-derived neurotrophic factor (MANF). This leads to the states of microglia from an iNOS-expressing to an Arg-1-expressing state (Yang et al., 2020). MANF is an inducible protein of endoplasmic reticulum stress and prevents cellular damage caused by such stress (Xu et al., 2019). The application of MANF treatment improved behavioral recovery in a rat model of stroke (Mätlik et al., 2018). In an *in vitro* model study using oxyhemoglobin-stimulated BV2 cells to simulate subarachnoid hemorrhage, it was found that microglia were cocultured with BMSCs, and BMSCs caused significant activation of the FoxO, TNF, and PI3K-Akt signaling pathways in microglia through cytokine secretion. Another study found BMSCs may inhibit the NF-κB and promote the PI3K/AKT signaling pathway by secreting glial cell-derived neurotrophic factor (GDNF) to inhibit LPS-induced expression of IL-1β, TNF-α, and CD86 and up-regulate the expression of IL-10, CD206 in microglia (Zhang et al., 2020; Zhong et al., 2020a,b). Yin et al. (2023) isolated multilineage-differentiating stress-enduring (Muse) cells from bone marrow mesenchymal stem cells. By co-culturing Muse cells with lipopolysaccharide-stimulated microglia, the results showed that Muse cells inhibited the expression of p38 mitogen-activated protein kinase (MyD88) and toll-like receptor 4 (TLR4) in microglia through paracrine effects. It also inhibited the phosphorylation of p38 MAPK, NF-κB inhibitor alpha, and transcription factor p65 in microglia, increasing the ratio of microglia expressing CD206 and Arg-1. That is, Muse cells produce anti-neuroinflammatory effects by inhibiting the TLR4/MyD88/NF-κB and p38 MAPK signaling pathways in microglia.

In a rat model of middle cerebral artery occlusion-induced brain injury, human umbilical cord-derived mesenchymal stem cells (hUC-MSCs) were found to migrate into the region of cerebral infarct and secrete various soluble cytokines, including prostaglandin E2 (PGE2). The ratio of expressing CD206 microglia in the brain area was significantly increased after hUC-MSCs (Teng et al., 2021). It has been shown that abnormalities in the immune-inflammatory pathway contribute to the development of major depression (Fenn et al., 2014), and inhibiting the overreactive state of microglia in response to inflammation facilitates the reduction of depressive-like behaviors. hUC-MSCs alter microglial states by inhibiting the C3a-C3aR signaling pathway through the secretion of C3a receptor antagonist-like substances, resulting in a decrease in hippocampal regions expressing CD16 + /Iba1 + microglia and an increase in the number of CD206 + /Iba1 + cells (Li et al., 2020).

In addition to BMSCs, hUC-MSCs and adipose-derived stem cells (ADSCs) have also been shown to regulate microglial states (Table 2). Nrf2-ADSC and BV2 cells were cocultured in the LPS environment. The results showed that ADSCs could increase Nrf2/HO-1 signaling pathway and decrease TLR4 and NF-κB signaling pathway activation in BV2 cells. As a result, BV2 cells expressed reduced levels of inflammatory cytokines such as MCP-1, TNF-α, IL-1β, and IL-6. Meanwhile, Nrf2-ADSC treatment also promoted the states of microglia toward the state of expressing Ym-1 and Arg-1 (Huang et al., 2020).

**TABLE 2:** MSCs regulate microglial states through cytokines.

Cell type	Cytokines	Function	References
MSCs	Gal-1, TSG-6, IL-13	Downregulate CD86^+^ and upregulate CD206^+^ microglia	Liu et al., 2014, 2019; Taj et al., 2018; Nam et al., 2020; Seo et al., 2022
BMSCs	PDGF-AA, GDNF	Downregulate CD86^+^ microglia	Yang et al., 2020; Zhong et al., 2020a,b
hUC-MSCs	PGE2, C3a receptor antagonist-like substances	Downregulate CD86^+^ and upregulate CD206^+^ microglia	Li et al., 2020; Teng et al., 2021
ADSCs	Nrf2	Downregulate CD86^+^ and upregulate CD206^+^ microglia	Huang et al., 2020

## MSCs regulate microglial states through their secreted extracellular vesicles

Extracellular vesicles are produced within the Golgi body and then released outside the cell through the merger of the Golgi body with the plasma membrane. Extracellular vesicles mediate intercellular communication by transferring proteins, miRNAs, and mRNAs (Kalluri and LeBleu, 2020).

Brain contusion with traumatic subarachnoid hemorrhage is a common clinical presentation in traumatic brain injury. The inflammatory response has been shown to be present throughout subarachnoid hemorrhage. It has been shown that MSCs significantly suppress the inflammatory response after subarachnoid hemorrhage by transferring extracellular vesicles (Han et al., 2021). Further studies revealed that MSCs-derived extracellular vesicles (MSCs-EV) inhibit NF-κB activation via activation of AMPK via its downstream proteins sirtuin-1 and Forkhead Box O3, thereby reducing the mRNA levels of IL-1β, CD11b, CD16, and iNOS and increasing the mRNA levels of TGF-β, Arg-1 and CD20 in microglia (Han et al., 2021). MSCs transfer miR-140-5p into microglia via extracellular vesicles. MiR-140-5p targets the activin-like kinase 5 gene and inhibits its expression, thereby downregulating NADPH oxidase 2 and thereby inhibiting CD86^+^ microglia activation in a mouse model of subarachnoid hemorrhage (Qian et al., 2022). Furthermore, extracellular vesicles miR-223-3p from MSCs attenuated cerebral ischemia/reperfusion injury by inhibiting the proinflammatory response mediated by microglial. MSCs promoted the conversion of microglia from the CD16/CD32^+^ to the CD206^+^ state, which may be related to the inhibitory effect of extracellular vesicles miR-223-3p on CysLT2R/ERK1/2 signaling pathway (Zhao Y. et al., 2020; Zhao Y. M. et al., 2020). Cysteinyl leukotrienes (CysLTs) are potent inflammatory mediators expressed after cell necrosis, and their inflammatory effects are mediated by the CysLTs receptor (Sasaki and Yokomizo, 2019). ERK1/2 are important mitogenic kinases in the ERK signaling pathway involved in the inflammatory response (Zhao Y. et al., 2020). MSCs also transfer miR-21a-5p via extracellular vesicles and repress the STAT3 gene, inducing microglia to shift from a proinflammatory to an anti-inflammatory state (Xin et al., 2022). Microglia play an important role in mediating CNS demyelinating diseases and their repair, while the generation of microglial reaction secondary to TBI is also dominated by microglial activation, and the treatment of both involves the regulation of microglial states. MSCs reduce TLR2/IRAK1/NF-kB signaling pathway activation in microglia by transferring extracellular vesicles, which in turn promote microglial conversion from the iNOS^+^ to the Ym-1^+^ (Zhang J. et al., 2022). Let-7a, miR-23a, miR-125b, and miR-199 in extracellular vesicles may play an important role in this process (Ferguson et al., 2018; Fan et al., 2020). In the treatment of a mouse model of spinal cord injury, it was found that MSCs pretreated with melatonin could promote the conversion of mouse microglia by secreting extracellular vesicles. The number of microglia expressing iNOS, TNF-α, and IL-1β was reduced, and the number of microglia expressing Arg-1, CD206, and Ym-1 was increased (Liu et al., 2021). Ubiquitin-specific protease 29 (USP29) in extracellular vesicles interacts with nuclear factor-like 2 in microglia, thereby regulating microglial states (Liu et al., 2021). In a model applying MSCs to treat spinal cord injury, it was found that extracellular vesicles miR-216a-5p secreted by hypoxia-pretreated MSCs are involved in the regulation of microglial states from the Iba-1^+^/iNOS^+^ to the Iba-1^+^/Arg-1^+^ state by regulating the TLR4/NF-kB/PI3K/AKT signaling pathway, namely, by inhibiting TLR4/NF-κB and activating the PI3K/AKT signaling pathway (Liu et al., 2020). More interestingly, the surface receptor type of extracellular vesicles also affects microglial states. MSCs influenced the states of microglia *in vivo* by secreting extracellular vesicles expressing the C-C chemokine receptor type 2 surface receptor, which may be a result of the binding of CCR2-expressing extracellular vesicles to endogenous C-C chemokine ligand 2 ligands that inhibit exogenous macrophage hypertropia and therefore suppress subsequent inflammatory cell states (Yang et al., 2020).

Extracellular vesicles secreted by human BMSCs (BMSCs-EV) reduce the number of CD86^+^ microglia by inhibiting the P38MAPK/P65NF-κB signaling pathway (Shu et al., 2022). A controlled cortical impact method was used to induce a mouse model of TBI, and the BMSCs-EV were injected through retro-orbital, which showed a decrease in the expression of pro-inflammatory cytokines (IL-1β, TNF-α) and an increase in the expression of anti-inflammatory proteins (Arg-1). The BMSCs-EV promotes the transformation of microglial cells of the Iba-1^+^/iNOS^+^ to the Iba-1^+^/CD206^+^ state (Ni et al., 2019). In a therapeutic model of cerebral ischemia-reperfusion, conditioned medium from hypoxia-pretreated BMSCs was found to promote anti-inflammatory states of microglia, and the exocytosis of these effects was mainly attributed to the extracellular vesicles secreted by BMSCs (Yu et al., 2021). In a rat model of middle cerebral artery occlusion, BMSCs were found to transfer miR-23a-3p via extracellular vesicles to downregulate the number of iNOS^+^ cells and upregulate the number of Arg-1^+^ cells (Dong et al., 2022). Lipopolysaccharide-stimulated BV2 microglia are widely accepted as a common *in vitro* model of microglial reaction. The secretion of extracellular vesicles H19 by BMSCs alleviated LPS-induced expression of IL-1β, IL-6, and TNF-a and increased the levels of IL-10 by targeting miR-29b-3p expression in microglia (Zong et al., 2021). Duan et al. (2020) found that BMSCs, by secreting extracellular vesicles miR-146a-5p, interact with interleukin-1 receptor-associated kinase 1 and nuclear factor of activated T cells 5 in microglia, thereby inhibiting the proinflammatory states of microglia after cerebral hemorrhage in rats. The development of multiple sclerosis also involves the states of inflammatory microglia in the CNS. In a study of a rat model of experimental autoimmune encephalomyelitis, Zi found that extracellular vesicles secreted by BMSCs inhibited the expression of the M1 phenotypic markers CD68, iNOS, and TNF-α in microglia and promoted the expression of the M2 phenotypic markers CD206, Arg-1, IL-10, and TGF-β (Li et al., 2019).

In a study of a rat model of TBI, it was found that the expression of pro-inflammatory factors was reduced by intracerebroventricular injection of human adipose mesenchymal stem cells (hADSCs)-derived extracellular vesicles (hADSCs-EV). Further studies found that hADSCs-EV inhibited the morphological transformation from the resting to the amoebic appearance of microglia by inhibiting the activation of NF-κB and P38/MAPK signaling pathways (Chen Y. F. et al., 2020). In a study of a mouse model of middle cerebral artery occlusion, ADSCs pretreated by hypoxia were found to transfer circ-Rps5 via extracellular vesicles. MiR-124-3p interacts with the Sirtuin 7 (SIRT7) 3′UTR. Overexpression of circ-Rps5 downregulates miR-124-3, which in turn promotes the expression of SIRT7 and converts microglia from the iNOS^+^ to the CD206^+^ state (Yang et al., 2022). SIRT7 and miR-124-3p are downstream targets of circ-Rps5 (Yang et al., 2022). MiR-124-3p inhibits the activation of nucleotide-binding oligomerization domain-like receptor pyrin domain-containing 3 inflammatory vesicles by targeting tumor necrosis factor receptor-associated factor 6, thereby suppressing microglial activation after brain basal ganglia hemorrhage cells secondary to microglial reaction (Fang and Hong, 2021). SIRT7 is an NAD + -dependent deacetylase that is involved in a variety of biological processes in organisms. In addition to mediating ribosomal RNA synthesis and protein synthesis, tumorigenesis, DNA damage response, and metabolism, SIRT7 can also inhibit LPS-induced inflammatory responses through the NF-κB signaling pathway (Chen et al., 2019). The extracellular vesicles secreted by ADSCs (ADSCs-EV) pretreated with hypoxia are enriched with LncGm37494, which promotes the states of microglial from the Iba-1^+^/iNOS^+^ to the Iba-1^+^/Arg-1^+^ state by inhibiting miR-130b-3p expression in microglia. ADSCs-EV also promote the expression of peroxisome proliferator-activated receptor γ (PPARγ), which in turn repairs spinal cord injury (Shao et al., 2020). PPARγ belongs to a family of nuclear receptors that was shown to polarize macrophages to the M2 phenotype (Yao et al., 2018). In addition, ADSCs inhibit ischemia-induced autophagy by secreting extracellular vesicles miR-30d-5p which targets the Beclin-1 and Atg5 genes. Consequently, ADSCs can inhibit the expression of inflammatory cytokines by microglia, promoting the states of microglia into a state that expresses anti-inflammatory cytokines such as IL-4 and IL-10 and alleviating neurological damage caused by cerebral infarction (Jiang et al., 2018).

In a model of retinal ischemia/reperfusion injury, TNF-α stimulated extracellular vesicles miR-21-5p secreted by gingival mesenchymal stem cells (GMSCs) was found to convert microglia from the Iba-1^+^/CD86^+^ state to the Iba-1^+^/Arg-1^+^ state by combining with programmed cell death 4 (PDCD4) (Yu et al., 2022).

In summary, MSCs mainly modulate inflammatory pathways after traumatic CNS injuries and promote the states of activated microglia in an anti-inflammatory direction (Table 3). Among them, BMSCs are easily isolated and expanded *in vitro*, in relatively abundant supply, and without ethical issues, making them ideal candidates for cell transplantation for inflammation suppression and cellular repair after various brain injuries, including TBI and spinal cord injury (Ning et al., 2019). For example, Bansal et al. (2016) successfully extracted bone marrow mesenchymal stem cells in patients and treated them with injections at the L1/L2 level. In a clinical trial of 10 patients with spinal cord injury, improvements in ASIA grading were observed in 6 patients, bladder control in 3 patients, sexual function in 5 patients, and relief of spasticity in 8 patients. Jarocha et al. (2015) conducted a 2-year trial of intensive treatment with bone marrow stem cell transplantation in a patient with a T2-3 spinal cord injury. After treatment, the patient’s ASIA classification increased from A to C/D, the sensory level increased from T1 to L3-4 and control of the body and urine was restored.

**TABLE 3:** The signaling pathway of extracellular vesicular miRNA secreted by MSCs to regulate microglia state.

Cell type	MiRNA or other	Signaling pathways	Function	References
MSCs	miR-140-5p	ALK5/NOX2	Downregulate CD86^+^ microglia	Qian et al., 2022
MSCs	miR-21a-5p	STAT3	Downregulate CD86^+^ and upregulate CD206^+^ microglia	Xin et al., 2022
MSCs	let-7a/miR-23a/miR-125b/miR-199	TLR2/IRAK1/NF-kB	Downregulate iNOS^+^ and upregulate Ym-1^+^ microglia	Zhang J. et al., 2022
MSCs	USP29	AMPK/NF-KB	Downregulate iNOS^+^/TNF-α^+^/IL-1β^+^and upregulate Arg-1^+^/CD206^+^/Ym-1^+^ microglia	Liu et al., 2021
MSCs	miR-223-3p	CysLT2R/ERK1/2	Downregulate CD16/CD32^+^ and upregulate CD206^+^ microglia	Zhao Y. et al., 2020
MSCs	miR-216a-5p	TLR4/NF-KB/PI3K/AKT	Downregulate Iba-1^+^/iNOS^+^ and upregulate Iba-1^+^/Arg-1^+^microglia	Liu et al., 2020
BMSCs	miR-23a-3p		Downregulate iNOS^+^ and upregulate Arg-1^+^ microglia	Dong et al., 2022
BMSCs	H19		Downregulate IL-6^+^/TNF-α^+^/IL-1β^+^ and upregulate iNOS^+^/TNF-α^+^/IL-1β^+^ microglia	Zong et al., 2021
BMSCs	miR-146a-5p		Downregulate CD86^+^ microglia	Duan et al., 2020
ADSCs	circ-Rps5	miR-124-3p/SIRT7	Downregulate iNOS^+^ and upregulate CD206^+^ microglia	Yang et al., 2022
ADSCs	LncGm37494	miR-130b-3p/PPARγ	Downregulate Iba-1^+^/iNOS^+^ and upregulate Iba-1^+^/Arg-1^+^ microglia	Shao et al., 2020
ADSCs	miR-30d-5p		Inhibit microglia expressing inflammatory cytokines	Jiang et al., 2018
hADSCs		NF-κB and P38/MAPK	Inhibit the morphological transformation of microglia from the resting to the amoebic appearance	Chen Y. et al., 2020
GMSCs	miR-21-5p	PDCD4	Downregulate Iba-1^+^/CD86^+^ and upregulate Iba-1^+^/Arg-1^+^ microglia	Yu et al., 2022

## Conclusion and perspectives nomenclature

All in all, BMSCs are the most accessible and widely used, while AMSCs can also be used in a range of brain injury diseases, all of which have a role in suppressing secondary inflammation response. MSCs implantation mainly regulates inflammatory pathways but also secretes certain cytokines or extracellular vesicles that affect apoptotic pathways, such as extracellular vesicles miR-21-5p, thus playing a role in neuronal protection.

How MSCs regulate macrophage states has been reported in the literature. In addition to the secretion of soluble cytokines and extracellular vesicles, MSCs can also regulate macrophage states through metabolic reprogramming, mitochondrial transfer, apoptosis, and phagocytosis. As intrinsic immune cells of the central nervous system, microglia may also be regulated in several of the above ways. In addition, MSCs from different sources have different roles. It has been reported that injection of allogeneic BMSCs improves neonatal hypoxic-ischemic brain injury, whereas ADSCs induce severe pulmonary hemorrhage and increased mortality (Sugiyama et al., 2018), which was not found in the abovementioned study. Whether these effects are related to the source of MSCs, the implantation method, or the implantation time, whether there are interactions between the pathways of action of MSCs secretions and how to improve the survival of implanted MSCs need to be demonstrated by further studies to better exploit the immunomodulatory function of MSCs.

## Author contributions

QX drafted the manuscript. WZ, CH, TL, and HW critically revised the manuscript. All authors contributed to the manuscript revision, read, and approved the submitted version.
